# MET transcriptional regulator/serine peptidase inhibitor kunitz type 1 panel operating through HGF/c‐MET axis as a prognostic signature in pan‐cancer

**DOI:** 10.1002/cam4.3834

**Published:** 2021-03-09

**Authors:** Yi Xiang, Bishan Liang, Yu Jiang, Fei Sun, Yang Zhao, Qijing Wu, Xingbin Hu, Yajing Liu, Qiong Huang, Wangjun Liao, Zhiqi Yao, Shaowei Li, Min Shi

**Affiliations:** ^1^ Department of Oncology Nanfang Hospital Southern Medical University Guangzhou China

**Keywords:** cell proliferation, HGF/c‐Met axis, MACC1, pan‐cancer, prognosis, SPINT1

## Abstract

Dysregulations in transcription factors (TFs) and their genetic products play important roles in tumorigenesis, tumor progression and metastasis. However, prognostic value of the transcriptional regulatory networks in different cancers has not been investigated in depth. The purpose of our study was to identify and validate a potential predictive signature that combines TFs and their regulatory products in eight solid tumors. We used bioinformatics analysis to identify MET Transcriptional Regulator (MACC1) and Serine Peptidase Inhibitor Kunitz Type 1 (SPINT1) as candidate TFs with the respective downstream regulatory proteins for patient prognosis in pan‐cancer. Subsequent molecular analysis of clinical gastric cancer tissue samples further verified the negative correlation between MACC1 and SPINT1. Further, we showed that mechanistically, MACC1/SPINT1 mediated the pro‐HGF proteolysis and c‐Met phosphorylation in HGF/c‐MET signaling pathway. Kaplan‐Meier plots and receiver operating characteristics analysis revealed that the two‐gene signature combining MACC1 with SPINT1 was effective in predicting survival in all eight cancer cohorts tested. In conclusion, our study clarified the regulatory relationship between MACC1 and SPINT1 in the context of the HGF/c‐MET signaling pathway and determined MACC1/SPINT1 panel as a valuable signature for the prediction of prognosis in patients for multiple solid cancer types.

## Graphical abstract

The manuscript addressed on the regulatory relationship and tumor prognostic value of transcription factors (TFs) MACC1 and transmembrane protein SPINT1. Through bioinformatics analysis and molecular experiments, we verified the transcriptional regulatory relationship and biological functions of MACC1 and SPINT1 in HGF/c‐Met signaling axis. Besides, MACC1/SPINT1 panel was established to effectively predict survival in eight cancers.

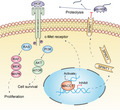

## 1 INTRODUCTION

Transcription factors (TFs) are key proteins that regulate transcription rate of mRNAs of specific genes by binding to the promoter sequences with a unique structure.^1^, ^2^ Previous studies have revealed that dysregulation of tumor‐associated TFs mediated several malignant biological processes, including uncontrolled cell proliferation, apoptosis, metastasis, and invasion by affecting the proper expression of downstream signaling molecules.^3^, ^4^, ^5^ Therefore, the expression levels of TFs and their regulatory products (e.g., mRNAs, proteins, miRNAs, and lncRNAs) have been investigated to establish strategies for cancer diagnosis, prognosis, and evaluation of therapeutic responses.^6^, ^7^, ^8^ As a result, several candidate TFs associated with tumor progression were identified.

It has been reported that MACC1 acts as a transcriptional regulator of proto‐oncogene c‐Met to promote proliferation, invasion, epithelial‐to‐mesenchymal transition, angiogenesis, and chemotherapy resistance in a variety of tumors through the activation of the downstream HGF/c‐Met/MAPK and HGF/c‐Met/AKT signaling pathways.^9^, ^10^, ^11^ An earlier study indicated that MACC1 was upregulated in gastric cancer (GC) causing elevated cell glycolysis under metabolic stress induced by nutrient deprivation during tumor progression.^12^, ^13^ Two other candidates, hepatocyte growth factor activator inhibitor 1 (HAI‐1) and a transmembrane inhibitor, serine peptidase Kunitz type 1 (SPINT1), inhibit multiple proteases activity, notably HGFA and matriptase.^14^ Mechanistically, SPINT1 blocks the conversion of inactive pro‐HGF to active HGF, which normally binds to c‐MET receptors and promotes c‐MET phosphorylation.^14^, ^15^, ^16^ Hence, SPINT1 has been reported as a pan‐cancer suppressor and a potential therapeutic target because of its inhibitory effect on carcinogenesis, invasion, and metastasis in esophageal,^17^ gastric,^18^ colorectal,^14^ breast,^19^, ^20^ lung,^21^ ovarian,^22^ cervical,^23^ prostate,^24^, ^25^ pancreatic,^26^, ^27^, ^28^ endometrial,^17^ renal,^29^ and oral squamous cell carcinoma cancers.^16^

The transcriptional regulation of membrane proteins is a common pattern of controlling physiological functions in eukaryotic cells.^30^ When deregulated, this transcriptional regulatory pattern plays a crucial role in the occurrence and progression of multiple cancers by mediating intracellular or intercellular signal transduction and regulating tumor‐associated downstream signaling pathways.^3^, ^4^, ^13^, ^31^ It was speculated that the above‐mentioned regulatory relationship between TFs and membrane proteins exists between MACC1 and SPINT1 because of their molecular characteristics. Even though both MACC1 and SPINT1 were validated to be associated with the HGF/c‐Met signaling pathway in multiple cancer types, synergistic regulations of MACC1 and SPINT1 in the context of the HGF/c‐Met signaling axis and the potential pan‐cancer prognostic value of these two gene combinations remain to be investigated.

This study aimed to identify and validate a potential predictive signature of TFs combined with their regulatory products in eight solid tumors. To overcome the complexity of transcriptional regulation, we applied integrated bioinformatics analysis to screen significant TFs in pan‐cancer prognosis, which was further validated through the molecular analysis of clinical samples. We focused on MACC1 and SPINT1, which were identified as important molecules for further investigation of their transcriptional regulatory relationship, biological functions in GC cells, and pan‐cancer prognostic value.

## 2 MATERIALS AND METHODS

### 2.1 Patients and tissue specimens

The use of human tissue specimens and clinical data was approved by the Nanfang Hospital Ethics Review Board (Guangzhou, China). The clinical cohort included, in total, 128 specimens of formalin‐fixed and paraffin‐embedded (FFPE) GC tissues from patients who were operated between January 2006 and December 2010, in Nanfang Hospital, Southern Medical University (Guangzhou, China). The enrolled patients were not treated with any preoperative therapy, and they were staged according to the criteria of the 8th Edition of the AJCC Cancer Staging Manual: Stomach (2017).^32^ Patients in stages I–III were evaluated on the basis of the disease‐free survival (DFS), and the others in stage IV were evaluated based on overall survival (OS). A written‐informed consent permitted by the Nanfang Hospital Ethics Review Committee (Guangzhou, China) was provided by all of the patients before the study.

### 2.2 Public dataset cohorts

The gene expression data and clinical information for eight cancer types (*n* = 20,241) were downloaded from The Cancer Genome Atlas (TCGA) database, a public comprehensive data repository of human cancer genome sequencing data.^33^ The overall data downloaded from the TCGA database was used to identify significantly up‐regulated and prognostic TFs among all eight cancer types. Moreover, five GC transcriptional expression datasets (GSE15459, GSE54129, GSE51105, GSE84437, and GSE62254) including 580 GC tissue samples and 579 non‐cancerous samples were obtained from the Gene Expression Omnibus (GEO) database, a free and open‐source functional genomics database covering a massive amount of high throughput gene expression data, chips, and microarrays.^34^ Next, all of the probes were transformed into symbols of the corresponding genes based on the annotation information.

### 2.3 Differential expression analysis

Five GC datasets from the GEO database were divided into subgroups with high and low expression levels of MACC1 according to the median RS cutoff. The differentially expressed genes (DEGs) between subgroups with high/low MACC1 were screened and overlapped among the five GC cohorts from GEO database. The R package limma was used to employ the Bayes method and build the linear model. The differences were considered to be significant for genes with fold change >1.5 and adjusted *p* values < 0.01.

### 2.4 Functional enrichment characterization of DEGs

Kyoto Encyclopedia of Genes and Genomes (KEGG) and gene ontology (GO) analyses for DEGs were accomplished with the OmicShare, which is an online data analysis platform (http://www.omicshare.com/tools), in order to explore the biological significance. Furthermore, gene set enrichment analysis (GSEA), a Java‐based powerful tool for interpreting the biological meaning of the DEGs, was also used to identify significantly enriched molecular signaling pathways. The GSEA procedure investigated whether a definite set of genes associated with a specific molecular signaling pathway was altered considerably between high and low MACC1 subgroups. Subsequently, an enrichment score for each set of genes was computed, which represented the overrepresentation degree of a gene set at both ends of the continuum.

### 2.5 Protein‐protein interaction (PPI) network construction and module analysis

Search Tool for the Retrieval of Interacting Genes (STRING) database^35^ was used to construct the PPI network of DEGs. The protein interaction analysis might facilitate the identification of gene sets that are associated with tumor progression. The molecular interaction networks were visualized using Cytoscape (Version 3.4.0), which is a software platform for bioinformatics data presentation.^36^ We used a plug‐in called Molecular Complex Detection (MCODE; Version 1.4.2) of Cytoscape, which is used for clustering a given network on the basis of topology, to find densely connected regions.^37^ The visualization of PPI networks was established using Cytoscape, and MCODE was used for the identification of the most significant module.

### 2.6 Co‐expression analysis in network databases

Co‐expression of MACC1 was analyzed using three network gene co‐expression search and visualization databases: SEEK,^38^ Coexpedia,^39^ and Oncomine tumor database.^40^ These co‐expression databases contain a massive amount of array‐based transcriptomics data that have been deposited in several public depositories such as GEO and ArrayExpress. They provide data mining ways for analyzing massive human expression compendium which currently contains thousands of expression datasets and returns a robust ranking of co‐expressed genes in the biological area depending on the users’ interest. The overlap between the screening results in the three databases was applied to identify the downstream signaling proteins regulated by MACC1.

### 2.7 Cell culture, plasmid construction, and transfection

Two human GC cell lines with c‐Met amplification (MKN45 and Hs746T) were obtained from Shanghai Foleibao Biotechnology Co. MKN45 cell line was cultured in RPMI 1640 medium and Hs746T in DMEM medium, both of which were supplemented with 10% fetal bovine serum (HyClone). The cells were maintained at 37°C under 5% CO2. For ectopic MACC1 overexpression, MACC1 coding sequence was amplified using PCR and cloned into the pBaBb‐puromycin plasmid (Obio Technology). The primer sequences were as follows: F‐CCGCTCGAGATGCTAATCACTGAAAGAAAAC, R‐CGCTCGAGCTATACTTCCTCAGAAGTGGAGAAT. To silence MACC1 expression, sequences of short hairpin RNA targeting MACC1 were cloned into the pSUPERretro‐puromycin plasmid. The two shRNA sequences used in this study were as follows: shMACC1#1 (5ʹ‐GCTGCCACCATTTGGGATT‐3ʹ), shMACC1#2 (5ʹ‐GCCCGTTGTTGGAAATCAT‐3ʹ). Subsequently, the overexpression and silencing plasmids were stably transfected into MKN45 and Hs746 T cell lines. Stable cell lines were selected with 1 μg/ml puromycin medium (Invitrogen) for 48 h after infection. Furthermore, to downregulate SPINT1 expression, small interfering RNAs (siRNAs) targeting SPINT1 were transiently transfected with Lipofectamine 2000 (Invitrogen). The siRNA sequences used in the two GC cell lines were siSPINT1#1 (5′‐CUGCAAGAGUUUCGUUUAU‐3ʹ), si‐SPINT1#2 (5′‐UUGACGAGCUCCAGCGC AU‐3ʹ), and siSPINT1#3 (5′‐GAACAACUACCUUCGGGAA‐3ʹ). A scrambled siRNA with disrupted synthesis sequence was used as a negative control.

### 2.8 Isolation of RNA and quantitative RT‐PCR analysis of gene expression

TRIzol kit was used to extract total RNA from GC cells. Quantitative RT‐PCR (qRT‐PCR) with SYBR Green dye (Takara, Japan) to measure gene expression was performed on the LightCycler 480 System (Roche, Penzberg). The primer sequences were MACC1 (F: ATCCGCCACAGATGCTTAA, R: CTTCAGCCCCAATTTTCATC); SPINT1 (F: TTGGAATTCGCGATGGCCCCTGCGAGGAC, R: TTAGACTCAGAGGGGCCGGGTGGTGT); GAPDH (F: ACCCAGAAGACTGTGGATGG, R: TCTAGACGGCAGGTCAGGTC).

### 2.9 Western blot analysis of protein expression

GC cells were washed with low‐temperature PBS and then homogenized in lysis buffer containing protease inhibitors (KeyGEN, Nanjing, China) on ice. After centrifugation, the supernatant containing the protein was collected. A 4 × SDS loading buffer was added to the supernatant containing total protein and boiled at 100°C for 10 min. Protein samples were separated on 10–15% SDS‐polyacrylamide gel using electrophoresis and then transferred onto polyvinylidene fluoride membranes, which were subsequently blocked for 1.5 h at room temperature with 5% skim milk supplemented with 0.1% Tween 20 (TBST). Each of them was incubated 20 h with a primary antibody (1:1000) at 4°C and then with a secondary antibody for 60 min at room temperature. A chemiluminescence (ECL) detection system was utilized to visualize the immunoreactive bands.

### 2.10 Immunohistochemical staining

Immunohistochemical (IHC) staining was performed with the Dako Envision System (Dako, Glostrup, Denmark) to detect the expression of MACC1 and SPINT1. A semi‐quantitative method was adopted to score protein expression levels in tumor tissues. In brief, based on the staining intensity, sections were scored as 0 for negative, 1 for weak, 2 for medium, and 3 for strong, wherein the staining extent was scored in the light of the area percentages: 0 (0%), 1 (1–25%), 2 (26–50%), 3 (51–75%) or 4 (76–100%). The product of the staining intensity and extent scores were the final staining scores (range 0–12) for the expression of MACC1 and SPINT1. Further analysis was performed by defining 0–2 as negative expression, 3–7 as low expression, and 8–12 as high expression.

### 2.11 Cell viability analysis

After cell transfection, 4000MKN45 and Hs746T cells were seeded in six replicates in 96‐well plates and incubated for 48 h. Then, 5 mg/ml 3‐(4,5‐dimethylthiazol‐2‐yl)‐2,5‐diphenyltetrazolium bromide MTT (Invitrogen) was added into each well (20 µl per well). After the incubation for 4 h at 37°C in the dark, 150 µl/well dimethyl sulfoxide (DMSO) was added to the cells. Measurement of the absorbance intensity was conducted at 570 nm using a microplate reader (Bio‐Rad). The percentage of cell viability was calculated as follows: % cell viability = ([mean absorbance in test wells]/[mean absorbance in control wells]) × %.

### 2.12 Colony formation assay

MKN45 and Hs746T cells were seeded in 12‐well plates at 1 × 10^3^ cells per well and cultured for 2 weeks. Then, the cells were washed twice with PBS, fixed with paraformaldehyde, and stained with 0.5% crystal violet. The plates were washed again and then photographed under a microscope.

### 2.13 Flow cytometry analysis of apoptosis

Cell apoptosis was measured by double staining with Annexin V‐Light 650/PI detection kit (Key Gen Bio TECH) using flow cytometry analysis (BD Biosciences) equipped with a Cell Quest software (BD Biosciences).

### 2.14 Prediction of miRNA regulatory mechanism

The candidate miRNA binding to SPINT1 was explored using TargetScan, an online prediction database of microRNA biological targets.^41^ PROMO3.0 database^42^ was used to search for potential TF binding sites in the promoter regions of specific miRNA (usually upstream from −2000 to −1 bp).

### 2.15 Statistical analysis

SPSS Version 22.0 software (SPSS) or R software (Version 3.3.2) were used for statistical analysis. Experimental groups’ differences were evaluated using Student's *t* test or one‐way analysis of variance (ANOVA). Chi‐squared and Mann‐Whitney tests were applied appropriately to assess the relations between MACC1 and SPINT1 expressions and clinicopathological parameters. The correlation index of SPINT1 and MACC1 expression levels in tumor tissue staining was analyzed using Spearman's correlation. The survival curves of individual groups were compared using the log‐rank test. Univariate and multivariate analyses were performed to determine the influence of risk factors on patient survival using Cox regression analysis. The reported results covered hazard ratios (HRs) and 95% confidence intervals (CIs). Curve analysis of time‐dependent receiver operating characteristic (ROC) was utilized to evaluate the predictive performance of MACC1 and SPINT1 in comparison with the signature based on these two genes. The area under the curve (AUC), ranging from 0.5 (for an uninformative marker) to 1 (for a perfect predictive marker), was a measurement for how well patient survival can be predicted with the gene signatures. All values were expressed as mean ± SD, and statistical significance was deemed as *p* < 0.05 (^*^ *p* < 0.05, ^**^ *p* < 0.01, ^***^ *p* < 0.001).

## 3 RESULTS

### 3.1 The differential expression and prognostic value of MACC1 in eight cancers from TCGA database

**FIGURE 1:**
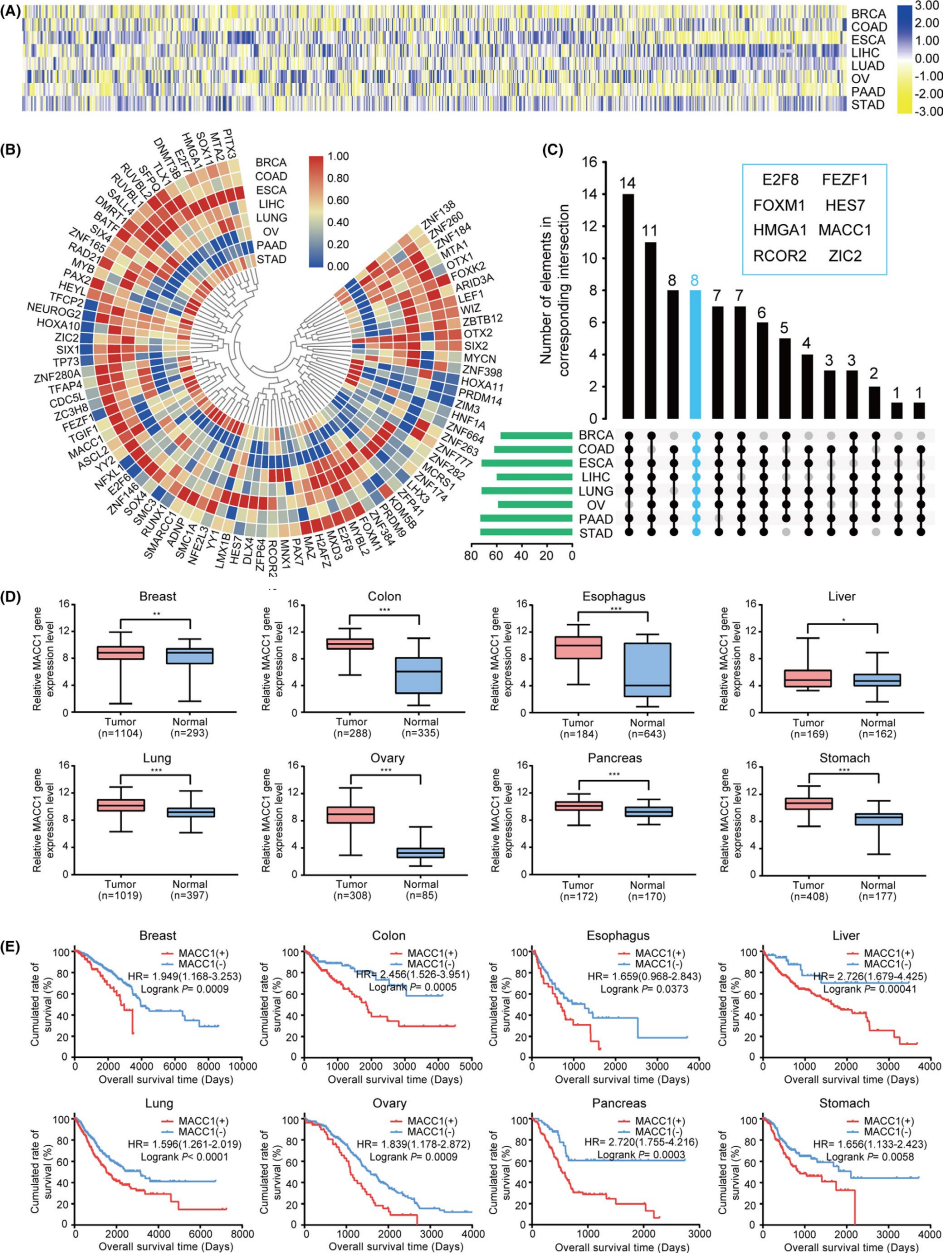
Differential expression analysis in eight tumors from TCGA database identifying MACC1 with prognostic value. A, Heatmap of the differential expressions of 896 TFs in eight cancer types from TCGA database. B, Circle heatmap of the standardized expressions of 88 TFs that screened from 896 TFs for the elevated expression levels in all the eight solid tumors. C, The upset‐plot displaying eight TFs with effective prognostic value in all the eight cancers. The *y* axis of the upper bar chart reflected the number of elements in each intersection, and the blue bar highlighted the only subset that overlaps in all eight cancer types, which contained eight TFs shown in the upper right box. Moreover, the bottom left bar chart in green showed the total amount of elements contained in each original dataset. D, RNA‐seq data showed that MACC1 expressions are upregulated in eight cancer tissues. E, Kaplan‐Meier analysis demonstrated that higher expression levels of MACC1 represented decreasing OS in patients with the eight cancers. ^*^ *p* < 0.05, ^**^ *p* < 0.01, ^***^ *p* < 0.001

First, the expression levels and survival predictive value of all of the 896 normal TFs in eight cancer types obtained from TCGA database were assessed to identify targeted TFs as potential biomarkers. Among the expression data for 20,530 mRNAs, expression values for the TFs were extracted and calculated using R (DESeq; Figure 1A). Gene expression levels between cancerous and each non‐cancerous tissues were compared to identify 88 TFs that were significantly upregulated in expression levels in all eight cancer patients (Figure 1B). Next, we evaluated the survival prognostic values for the 88 TFs and found that eight specific TFs (E2F8, FEZF1, FOXM1, HES7, HMGA1, MACC1, RCOR2, and ZIC2) were very effective in predicting the prognosis (Figure 1C). The blue bar of upset‐plot represented the intersection of all the eight cancer datasets, and HR calculated with Cox regression analyses showed in Table 1. The results shown above and our previous studies on MACC1 in GC and colorectal cancer patients demonstrated that MACC1 may be good at predicting the diagnosis and prognosis in patients with six other cancer types. As is shown in Figure 1D,E and S1, MACC1 showed significant difference in expression and predictive value of survival. In brief, MACC1 could be identified as a candidate prognostic biomarker and play a regulatory role as TF among the eight cancer types.

**TABLE 1:** Univariate Cox regression analysis for the eight transcription factors

No.	Gene symbol	Full name	HR (95%CI)	*p* value
1	E2F8	E2F Transcription Factor 8	1.153 (1.032–1.288)	0.012*
2	FEZF1	FEZ Family Zinc Finger 1	1.644 (1.359–1.989)	<0.001***
3	FOXM1	Forkhead Box M1	1.312 (1.175–1.466)	<0.001***
4	HES7	Hes Family BHLH Transcription Factor 7	1.224 (1.091–1.372)	0.004**
5	HMGA1	High Mobility Group AT‐Hook 1	1.525 (1.346–1.727)	<0.001***
6	MACC1	MET Transcriptional Regulator MACC1	1.643 (1.469–1.836)	<0.001***
7	RCOR2	REST Corepressor 2	1.302 (1.166–1.455)	<0.001***
8	ZIC2	Zic Family Member 2	1.385 (1.240–1.548)	<0.001***

Abbreviations: CI, confidence interval; HR, hazard ratio.

* *p* < 0.05.

** *p* < 0.01.

*** *p* < 0.001.

### 3.2 Identification and functional enrichment of DEGs regulated by MACC1

**FIGURE 2:**
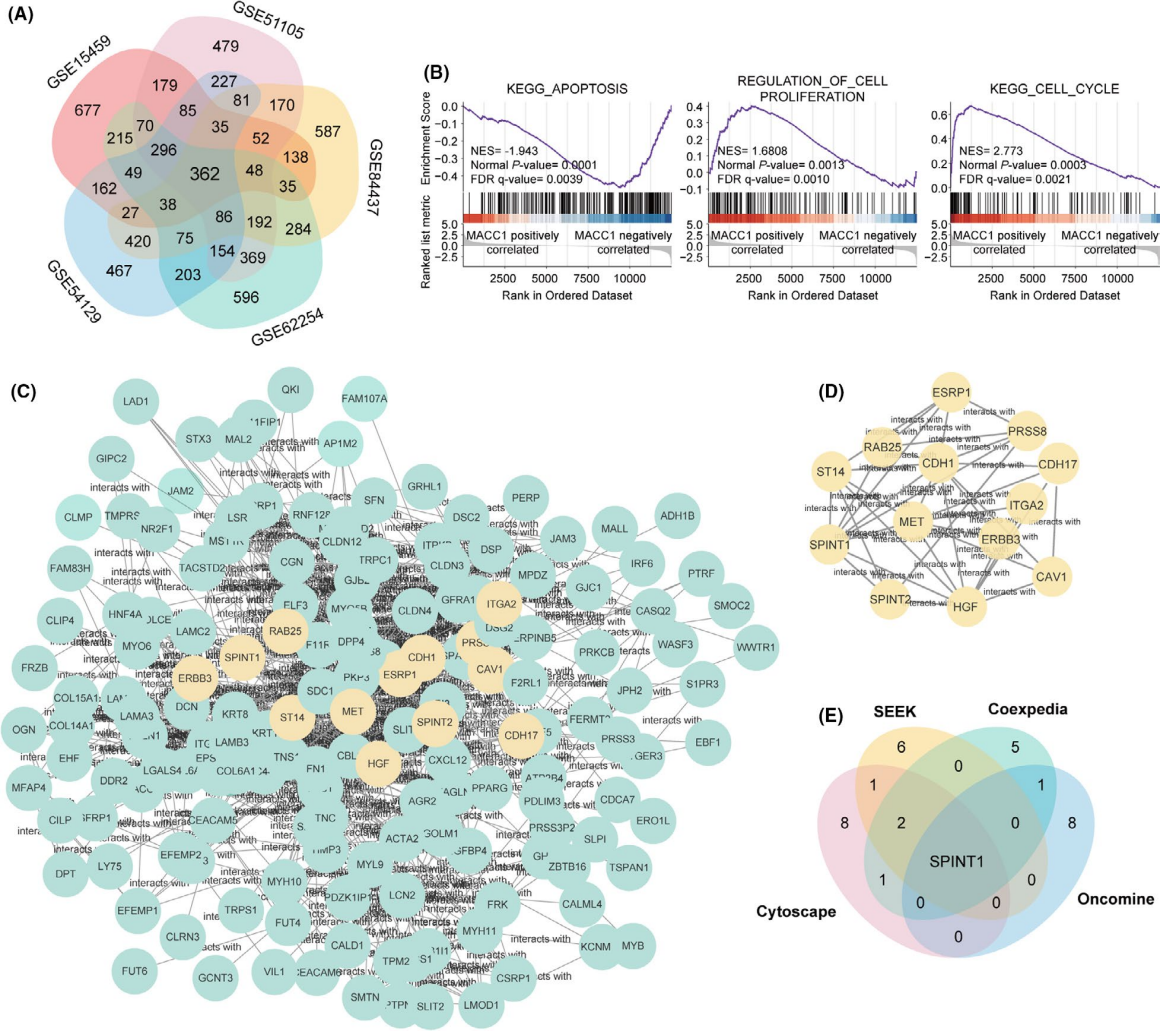
SPINT1 identified as the candidate downstream regulatory protein of MACC1 that was found to be related to proliferation and apoptosis of cell in functional enrichment analysis. A, Venn diagram displayed the intersection of DEGs among five GEO datasets of gastric cancer (GSE15459, GSE51105, GSE54129, GSE62254, and GSE84437). B, GSEA analysis showed that overexpression of MACC1 was involved in regulation of cell proliferation, cell cycle, and apoptosis. C, The PPI network of the 362 overlap DEGs established by Cytoscape. D, The most remarkable module gotten from PPI network consists of 13 hub genes marked in light yellow. E, Venn diagram identified SPINT1 as the only one hub gene correlatively expressed with MACC1 both in PPI network and three network co‐expression analysis databases

Five GC datasets from the GEO database were divided into subgroups with high and low MACC1 on the basis of the median RS cutoff. After the microarray data were standardized, DEGs (2,468 in GSE15459, 2,885 in GSE51105, 2,767 in GSE54129, 3,072 in GSE62254, and 2,630 in GSE84437) were identified. Venn diagram demonstrated the intersections of genes (362 DEGs) among the five datasets (Figure 2A), which consisted of 205 downregulated and 157 upregulated genes. Furthermore, the biological classification and characterization of the 362 DEGs were performed. Results of the KEGG pathway analysis revealed that the DEGs were mainly enriched in PI3 K‐Akt, Wnt, Rap1, cGMP‐PKG, cell cycle, apoptosis, and NF‐κB signaling pathways (Figure S2B). Tissue development, epithelial cell differentiation, cell adhesion, and cell junction organization were found to be the dominant cell functions identified in the GO analysis (Figure S2C). Further analysis of GSEA indicated that cell proliferation, cell cycle, and apoptosis were considered as the representative signaling pathways significantly enriched in the tissue samples of GC patients with highly expressed MACC1 (Figure 2B).

### 3.3 Module analysis based on the PPI network and screening co‐expressed genes with MACC1

The PPI network of DEGs was built with Cytoscape (Figure 2C), and the most significant module comprising a total of 13 hub genes was generated from the PPI network using the MCODE plug‐in (Figure 2D). The gene symbols are shown in Table 2. In addition, the genes significantly co‐expressed with MACC1 were further investigated among the three network co‐expression analysis databases SEEK, Coexpedia, and Oncomine. The top 10 genes whose expression was strongly correlated with MACC1 in the three databases are listed in Table 2. Eventually, the overlap analysis identified only one hub gene, the Serine Peptidase Inhibitor Kunitz Type 1 (SPINT1; Figure 2E). Considering these results and the previously published data, SPINT1 has a close predictive relationship with MACC1 and may have potential and vital functions downstream of MACC1 regulated GC progression.

**TABLE 2:** Top 10 genes co‐expressed with MACC1 in three co‐expression analysis databases

No.	SEEK		Coexpedia		Oncomine	
	Gene list	Score	Gene list	Score	Gene list	Correlation
1	PHLDB2	2209	RAB25	3.049	STX19	0.895
2	SH3D19	2.155	SPINT1	2.856	B3GNT3	0.895
3	TFPI	2.052	GRHL2	2.788	ANKRD22	0.79
4	SGMS2	1.988	LAD1	2.701	ATP2C2	0.79
5	EGFR	1.968	ST14	2.689	OVOL1	0.79
6	TNFRSF12A	1.944	EPHA1	2.669	BSPRY	0.781
7	SPINT1	1.936	PRSS8	2.637	ELMO3	0.776
8	PRSS8	1.907	KDF1	2.572	KRTCAP3	0.776
9	CAV1	1.875	OVOL1	2.563	SPINT1	0.768
10	RAB25	1.823	MARVELD3	2.515	CBLC	0.768

### 3.4 MACC1 negatively regulates the transcriptional level of SPINT1 in GC cells

**FIGURE 3:**
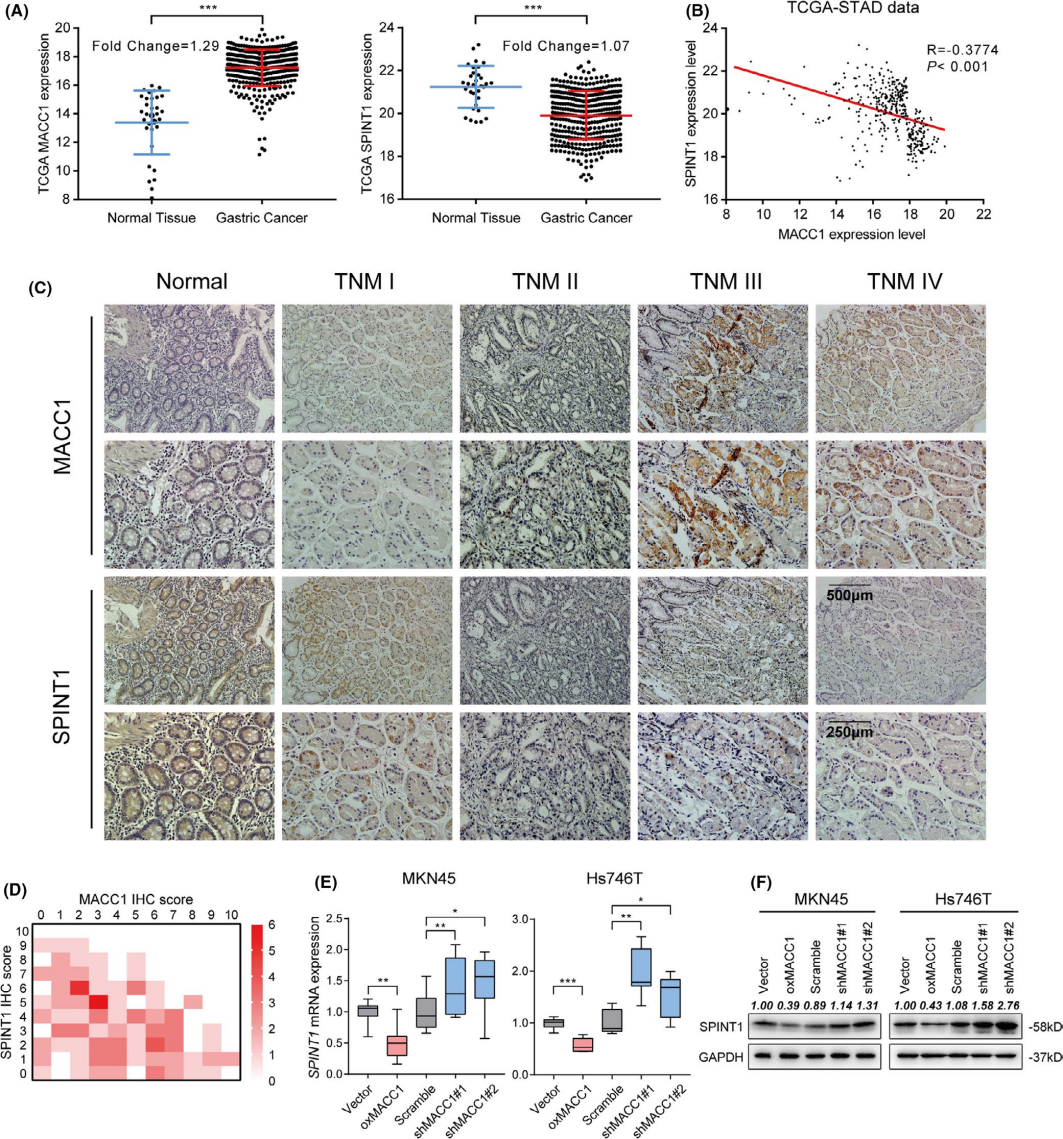
Expression of SPINT1 was negatively associated with MACC1 in GC tissues and transcriptionally suppressed by MACC1 in GC cells. A, RNA‐seq data from the TCGA database demonstrated the expressions of SPINT1 and MACC1 in GC tissues and non‐carcinoma counterparts. B, Pearson's correlation analysis showing the negative correlation between MACC1 and SPINT1 expressions. C, Representative successive IHC staining showed the expression density of MACC1 and SPINT1 in various TNM stages GC tissues and adjacent normal gastric mucosa tissues. D, Co‐expression heatmap showed that the expression level of SPINT1 was negatively related to MACC1 in cancer tissues and paired normal ones. The vertical axis shows SPINT1 scores, whereas horizontal axis shows MACC1 scores. The crossing gridiron indicated the quantity of cases (based on various concentrations of red color). E, mRNA expression levels of SPINT1 in MKN45 and Hs746T cells transfected with plasmids that stably overexpressed or silenced MACC1 (oxMACC1, shMACC1#1, and shMACC1#2) and their corresponding controls. F, The protein expression levels of SPINT1 in MKN45 and Hs746T cells by Western blotting. ^*^ *p* < 0.05, ^**^ *p* < 0.01, ^***^ *p* < 0.001

Although many previous studies have clarified that MACC1, which transcriptionally regulates MET expression, plays an important role in stimulating the c‐MET signaling pathway,^10^, ^43^, ^44^ to date, there was no study showing an association between MACC1 and SPINT1.^14^, ^16^ Therefore, the relationship between MACC1 and SPINT1, as well as their effect on proliferation and survival in MET‐amplified human GC cells, was verified further. As shown in Figure 3A, SPINT1 expression levels in GC patients from TCGA database were notably lower than those in normal tissues. Spearman's correlation analysis demonstrated a significantly negative relationship between MACC1 and SPINT1 (*r* = −0.3774, *p* = 0.025; Figure 3B). The GC patient prognosis was analyzed by categorizing the patients into two sets according to the expression level of MACC1 or SPINT1 using the online Kaplan‐Meier plotter analysis tool. The results demonstrated that lower expression of MACC1 or higher expression of SPINT1 was associated with prolonged OS and DFS (Figure S3A,B), which implied that both MACC1 and SPINT1 were associated with the prognosis in GC patients.

Furthermore, IHC was performed to measure the expression levels of the two genes in 128 pairs of GC samples (cancerous and the corresponding adjacent non‐cancerous tissues). The clinicopathological characteristics of these patients are shown in Table 3. The IHC results revealed that SPINT1 expression was higher in non‐cancerous than in GC tissue samples and the intensity and extent were decreased as the TNM stage advanced (Figure 3C). Meanwhile, the expression of MACC1 exhibited a negative correlation with SPINT1 according to the IHC staining (Figure 3C,D).

**TABLE 3:** Correlation between MACC1, SPINT1, and clinicopathological characteristics in gastric cancer patients

*n* (%)		MACC1				SPINT1			
		Low and negative (*n*)	High (*n*)	^c2^	*p*	Low and negative (*n*)	High (*n*)	^c2^	*p*
Age (years)				0.031	0.859			0.009	0.925
≥55	69 (53.9%)	35	34			38	31		
<55	59 (46.1%)	29	30			32	27		
Gender				0.133	0.715			0.210	0.647
Male	80 (62.5%)	39	41			45	35		
Female	48 (37.5%)	25	23			25	23		
TNM classification				17.929	0.001**			18.591	0.001**
I	8 (6.3%)	6	2			0	8		
II	23 (18.0%)	14	9			11	12		
III	79 (61.7%)	43	36			43	36		
IV	18 (14.1%)	1	17			16	2		
Tumor invasion				8.969	0.030*			18.780	0.001**
T1	8 (6.3%)	6	2			0	8		
T2	20 (15.6%)	15	5			6	14		
T3	39 (30.5%)	17	22			27	12		
T4	61 (47.7%)	26	64			37	24		
Lymph node metastasis				4.885	0.180			6.062	0.109
N0	39 (30.5%)	25	14			15	24		
N1	23 (18.0%)	10	13			14	9		
N2	38 (29.7%)	18	20			23	15		
N3	28 (21.9%)	11	17			18	10		
Distant metastasis				13.906	0.001**			7.848	0.005**
M0	109 (85.2%)	62	47			54	55		
M1	19 (14.8%)	2	17			16	3		
Tumor differentiation				3.675	0.159			5.207	0.074
Well	12 (9.4%)	5	7			10	2		
Moderate	40 (31.3%)	25	15			23	17		
Poor	76 (59.4%)	34	42			37	39		
Mortality				3.501	0.061			4.705	0.030*
Survive	111 (86.7%)	53	58			68	43		
Die	17 (13.3%)	4	13			15	2		
Recurrence				10.502	0.001**			3.201	0.074
No	38 (35.8%)	27	11			19	19		
Yes	68 (64.2%)	26	42			46	22		

* *p* < 0.05.

** *p* < 0.01.

To support the results obtained from the analysis of this relationship, we transfected MKN45 and Hs746T human GC cell lines with plasmids that were stably overexpressed (oxMACC1) or silenced MACC1 (shMACC1). The overexpression and silencing efficiency were evaluated for later functional studies (Figure S4A,B). Western blot and qRT‐PCR analyses showed that SPINT1 expression was suppressed by MACC1 in MKN45 and Hs746T cells (Figure 3E,F). Meanwhile, we tried to determine if a non‐coding RNA regulation mechanism plays a role in the altered expression of SPINT1 mediated by MACC1. Hsa‐miR‐183‐5p was identified as the microRNA that may potentially target SPINT1 (Figure S5A) with its upstream promoter region containing several potential binding sites of MACC1 (Figure S5C,D). Shortly, these results showed that MACC1 downregulated SPINT1 expression in GC cells and tissues.

### 3.5 The MACC1/SPINT1 axis regulates proliferation and apoptosis in GC cells via the downstream HGF/c‐Met signaling pathway

**FIGURE 4:**
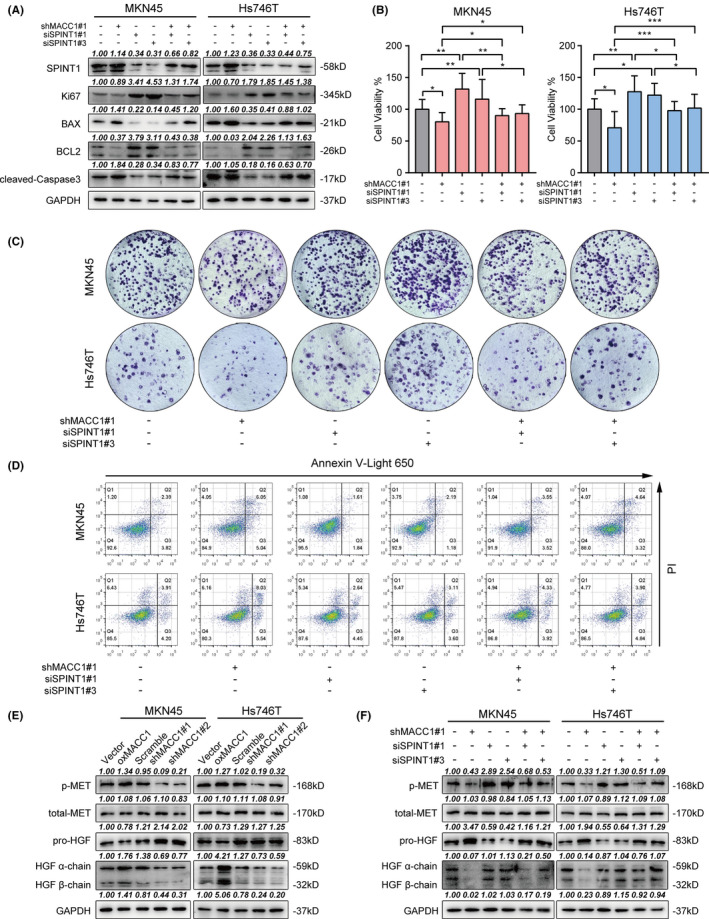
MACC1 regulates GC cell proliferation and apoptosis via the SPINT1/HGF/c‐MET axis. A, Western blot analysis showed the expression of apoptosis biomarkers Bax, cleaved‐caspase3, proliferation marker Ki67, and anti‐apoptosis protein Bcl‐2 in MKN45 and Hs746T cells co‐transfected with MACC1 shRNA and SPINT1 siRNA sequences. B, Effects of MACC1 and SPINT1 silencing on cell survival in MKN45 and Hs746T cells by MTT assay. C, The colony formation assay showing GC cell proliferation influenced by MACC1 and SPINT1 silencing. D, Flow cytometry analysis evaluating cell apoptosis rate in each group. E, Western blot analysis of representative proteins in HGF/c‐Met axis. The cleavage of pro‐HGF precursor was examined by anti‐HGF α/β chain antibody. F, SPINT1 knockdown partly reversed the decreased pro‐HGF proteolysis and c‐Met phosphorylation caused by MACC1 silencing. ^*^ *p* < 0.05, ^**^ *p* < 0.01, ^***^ *p* < 0.001

It had been reported that MACC1 regulated cell proliferation, apoptosis, and chemotherapy resistance in many tumors.^10^, ^45^, ^46^, ^47^, ^48^ We wondered whether SPINT1 was involved in this function of MACC1. To better understand the roles and mechanisms of SPINT1 action on the malignant biological processes of GC cells, we transiently transfected both MKN45 and Hs746T cell lines with SPINT1 siRNA sequences. As expected, the expression of SPINT1 was remarkably downregulated on both transcription and translation levels compared with NC group (Figure S6A,B). Next, the impact of MACC1/SPINT1 axis on GC cell proliferation and apoptosis was examined. Western blot assays showed that MACC1 overexpression suppressed the apoptosis biomarkers Bax and cleaved‐caspase3 and increased the expression levels of proliferation marker Ki67 and anti‐apoptosis protein Bcl‐2 in GC cells (Figure S4C). Meanwhile, MACC1 knockdown promoted the expression of cleaved‐caspase‐3 and Bax and inhibited the expression of Ki67 and Bcl‐2, which could be partly reversed by co‐transfection of SPINT1 siRNAs (Figure 4A). Promoted proliferation and colony formation capacity of MKN45 and HS746T were detected using MTT and colony formation assays in cells with overexpressed MACC1 (Figure S4D,E) and silenced SPINT1 (Figure 4B,C and S6C). Moreover, it was found that cell apoptosis induced by MACC1 knockdown was partly rescued after SPINT1 silencing (Figure 4D, S4F, and S6D).

Previous research verified that MACC1 regulated GC cell proliferation through HGF/c‐Met signaling pathway, which mainly depends on the pro‐HGF cleavage and c‐Met receptor phosphorylation levels.^43^, ^49^, ^50^ Meanwhile, SPINT1 inhibited the maturation of HGF via suppressing the activity of matriptase, the key enzyme for proteolysis, and activation of pro‐HGF.^14^, ^15^, ^16^ A Western blot assay was performed to examine the pro‐HGF proteolysis and c‐Met receptor phosphorylation level, so as to explore whether MACC1 promoted HGF/c‐Met signaling pathway partially by downregulating SPINT1 expression. The mature status and precursor status of HGF were detected by using anti‐HGF α/β chain antibodies and pro‐HGF antibodies,^19^, ^51^ respectively. In MKN45 and Hs746T cells, MACC1 knockdown reduced the proteolysis of pro‐HGF and c‐Met phosphorylation (Figure 4E), which was dramatically rescued by SPINT1 silencing in comparison with the scramble‐transfected cells (Figure 4F). In summary, our findings further demonstrated that MACC1 promoted pro‐HGF maturation and signal transduction in a SPINT1‐dependent manner, and MACC1 regulated cell proliferation and apoptosis via the downstream SPINT1/HGF/MET signaling axis in GC.

### 3.6 Prognostic value of MACC1 and SPINT1 expressions in GC patients

**FIGURE 5:**
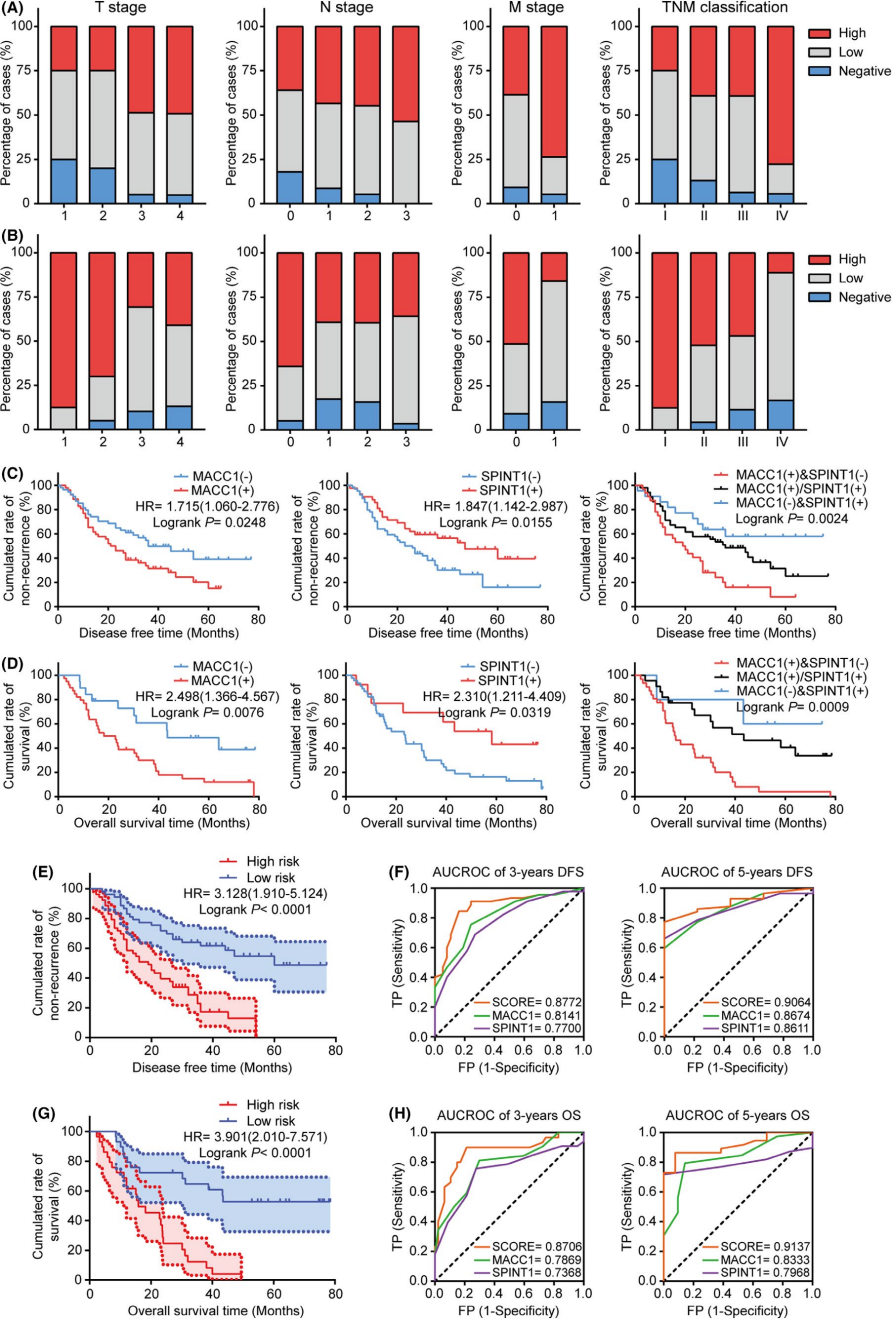
Prognostic values of MACC1 and SPINT1 in GC samples. A, B, Frequency of negative, low, and high MACC1 (A) and SPINT1 (B) expressions in GC samples classified by TNM stage. C, D, Kaplan‐Meier analysis of DFS (C) and OS (D) in GC patients. E–H, Kaplan‐Meier analysis and ROC curves showing the efficacy of the MACC1/SPINT1 prognostic signature on 3‐ and 5‐year DFS (E, F) and OS (G, H)

As given the negative relationship between SPINT1 and MACC1 in GC tissues and cells demonstrated above, we next evaluated their prognostic value relative to different clinicopathological factors. Figure 5A showed that compared with TNM stage, high MACC1 expression was observed at higher frequency in patients with the more advanced T stage (*p* < 0.05), N stage (*p* < 0.01), M stage (*p* < 0.05), and overall TNM stage (*p* < 0.05), while SPINT1 expression caused the opposite tendency (Figure 5B). Kaplan‐Meier survival analysis revealed that the DFS (stages I–III) and OS (stage IV) in GC patients with lower MACC1 expression levels were prolonged compared with those who had higher MACC1 expression (*p* = 0.0076, *p* = 0.0248; Figure 5C,D), while SPINT1 showed an opposite impact on these outcomes. Univariate and multivariate Cox regression analyses indicated that SPINT1 and MACC1 were independent prognostic factors for mortality and recurrence in GC patients (Tables 4 and 5). And Spearman analysis in Table 6 showed the links between the two genes and clinicopathological parameters. Briefly, our data suggested the potential combinatorial role of MACC1 and SPINT as a prognostic predictor for GC.

**TABLE 4:** Univariate and multivariate Cox regression analysis for recurrence in stages I–III GC patients

Variables	Univariate analysis		Multivariate analysis	
	HR (95%CI)	*p* value	HR (95%CI)	*p* value
I–III stages				
Age (≥55 vs. <55)	1.533 (0.934–2.517)	0.091	1.468 (0.879–2.452)	0.142
Gender (male vs. female)	0.805 (0.480–1.350)	0.411	1.056 (0.610–1.828)	0.845
TNM classification	2.337 (1.350–4.045)	0.002**	0.685 (0.261–1.803)	0.444
Tumor invasion	1.893 (1.382–2.594)	<0.001***	1.773 (1.068–2.943)	0.027
Lymph node metastasis	1.400 (1.132–1.732	0.002**	1.274 (0.965–1.682)	0.088
Tumor differentiation	1.073 (0.744–1.548)	0.704	0.925 (0.617–1.388)	0.708
MACC1 expression (high vs. low and negative)	2.470 (1.599–4066)	<0.001***	1.731 (1.030–2.910)	0.038*
SPINT1 expression (high vs. low and negative)	0.331 (0.198–0.553)	<0.001***	0.475 (0.266–0.847)	0.012*

Abbreviations: CI, confidence interval; HR, hazard ratio.

* *p* < 0.05.

** *p* < 0.01.

*** *p* < 0.001.

**TABLE 5:** Univariate and multivariate Cox regression analysis for mortality in stage I‐III and IV GC patients

Variables	Univariate analysis		Multivariate analysis	
	HR (95%CI)	*p* value	HR (95%CI)	*p* value
I‐III stage				
Age (≥55 vs. <55)	0.695 (0.281–1.719)	0.431	0.775 (0.235–2.551)	0.675
Gender (Male vs. Female)	0.981 (0.398–2.418)	0.996	0.469 (0.103–2.131)	0.327
TNM classification	2.349 (1.014–5.444)	0.046*	3.186 (0.232–43.659)	0.386
Tumor invasion	1.471 (0.928–2.333)	0.101	0.357 (0.065–1.951)	0.235
Lymph node metastasis	1.742 (1.126–2.694)	0.013*	0.872 (0.352–2.157)	0.766
Tumor differentiation	0.996 (0.462–2.148)	0.993	0.527 (0.150–1.858)	0.319
MACC1 expression (high vs. low and negative)	30.019 (4.779–302.427	0.001**	13.941 (1.187–163.736)	0.036*
SPINT1 expression (high vs. low and negative)	0.016 (0.002–0.130)	<0.001***	0.017 (0.001–0.385)	0.010*
IV stage				
Age (≥55 vs. <55)	0.892 (0.357–2.227)	0.807	1.299 (0.451–3.742)	0.627
Gender (Male vs. Female)	1.347 (0.535–3.388)	0.527	0.756 (0.235–2.436)	0.640
Tumor invasion	3.319 (1.165–9.451)	0.025*	1.819 (0.344–9.609)	0.481
Lymph node metastasis	1.926 (0.730–5.082)	0.185	0.880 (0.198–3.906)	0.867
Tumor differentiation	0.200 (0.046–0.873)	0.032*	0.306 (0.047–2.012)	0.306
MACC1 expression (high vs. low and negative)	22.482 (2.814–179.629)	0.003**	13.611 (1.233–150.235)	0.033*
SPINT1 expression (high vs. low and negative)	0.096 (0.024–0.384)	0.001**	0.257 (0.050–1.327)	0.105

Abbreviations: CI, confidence interval; HR, hazard ratio.

* *p* < 0.05.

** *p* < 0.01.

*** *p* < 0.001.

**TABLE 6:** Spearman analysis of correlation between MACC1, SPINT1, and clinicopathological parameters

Variables	MACC1 expression level		SPINT1 expression level	
	Spearman correlation	*p* value	Spearman correlation	*p* value
Age (years)	0.077	0.387	0.038	0.673
Gender	−0.108	0.223	0.026	0.775
TNM classification	0.300	<0.0001***	−0.271	0.002**
Tumor invasion	0.204	0.021*	−0.164	0.064
Lymph node metastasis	0.206	0.020*	−0.133	0.135
Distant metastasis	0.297	0.001**	−0.223	0.011*
Tumor differentiation	0.016	0.860	−0.179	0.044*
MACC1 expression	—	—	−0.396	<0.0001***
SPINT1 expression	−0.396	<0.0001***	—	—

* *p* < 0.05.

** *p* < 0.01.

*** *p* < 0.001.

The following Kaplan‐Meier survival analysis confirmed that the combination based on MACC1 and SPINT1 expression levels in a logistic regression model had better prognostic value of OS and DFS than the expression of MACC1 or SPINT1 separately (HR = 3.901 of OS, *p* < 0.0001 and HR = 3.128 of DFS, *p* < 0.0001; Figure 5E,G). Next, their efficacy in evaluating the survival time of GC was corroborated through ROC analysis. As shown in Figure 5H, AUCs for 3‐year OS for MACC1 and SPINT1 were 0.7869 and 0.7368, respectively, while those for 5‐year OS were 0.8333 and 0.7968, respectively. After SPINT1 and MACC1 were combined, the prognostic performance was improved compared with their individual efficacies (AUC = 0.8706 for 3‐year OS and 0.9137 for 5‐year OS). Similarly, the combination also displayed a better predictive performance for DFS (Figure 5F). Collectively, our results indicated that expression levels of MACC1‐SPINT1 panels used as a predictive model may enhance the prognostic value in GC patients.

### 3.7 Prognosis of the MACC1/SPINT1 signature in the eight cancers

**FIGURE 6:**
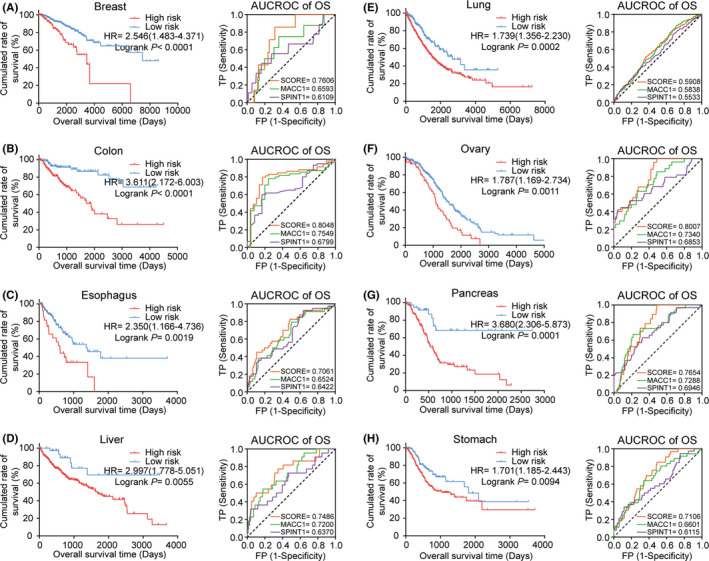
The pan‐cancer prognostic value of the MACC1/SPINT1 signature in eight cancer types. A–H, Kaplan‐Meier plots of OS in patients across eight cancers stratified into groups of low and high risks with the MACC1/SPINT1 prognostic signature. I–P, Time‐dependent ROC curve analysis of MACC1/SPINT1 signature for survival prediction in patients with the eight cancers

Kaplan‐Meier survival analysis was carried out in all of the eight cancer cohorts to assess the ability of the prognostic signature derived from GC to predict OS and DFS in other solid malignancies. Patients ranked by their scores were separated into high‐risk or low‐risk subgroups. Remarkably, the OS rates were drastically decreased in high‐risk subgroups among the eight cancer types: breast (HR = 2.546, *p* < 0.0001), colon (HR = 3.611, *p* < 0.0001), esophagus (HR = 2.350, *p* = 0.0019), liver (HR = 2.997, *p* = 0.0055), lung (HR = 1.739, *p* = 0.0002), ovary (HR =1.787, *p* = 0.0011), pancreas (HR =3.680, *p* = 0.0001), and stomach (HR =1.701, *p* = 0.0094; Figure 6A–H). Likewise, DFS was also remarkably decreased in the high‐risk subgroup (Figure S7A–H). Moreover, the predictive efficacy of the two‐gene prognostic signature on OS and DFS was performed by using the ROC curve analysis again in comparison with the expression levels of MACC1 and SPINT1 separately. As shown in Figure 6 and Figure S7, it was without exception that the AUCs for OS and DFS for the signature in the eight cancers were elevated, compared with those for MACC1 or SPINT1 separately, indicating that the MACC1/SPINT1 signature was an effectively prognostic model in these cancers.

## 4 DISCUSSION

Here, we performed a bioinformatics and molecular analyses of clinical samples for the identification of a MACC1/SPINT1 panel as a pan‐cancer prognostic tool. This panel was also determined its function in tumor progression via the HGF/c‐Met axis. Due to the limited gene signature studies on TFs and their downstream proteins in pan‐cancer, we hoped to further explore the biological effects and tumor prognostic value of key TFs.

Increasing amounts of evidence suggested that dysregulations of TFs are involved in multiple biological processes in cancers through the regulation of downstream signaling events.^2^, ^4^, ^13^ Increased attention has been paid to the guiding value of TFs and their regulatory products in cancer diagnosis and prognosis. For example, it has been reported that specific oncogenic TFs and miRNAs were screened to build predictive signature models for early diagnosis and prognosis of renal cell carcinoma based on TCGA database.^8^ In the studies on TF regulation, it is found that the interaction between TFs and membrane proteins is one of the important regulatory patterns. Regulated by specific TFs, the corresponding membrane proteins mediate the preparatory work of extracellular factors before binding to membrane receptors.^52^, ^53^, ^54^, ^55^ However, most studies hitherto performed only bioinformatics analysis on TFs and other functional proteins or non‐coding RNAs. Research on the predictive signature based on transcriptional regulatory relationship confirmed by molecular experiments is still limited. MACC1 was first described in 2009 as a critical pro‐metastatic TF regulating HGF/c‐Met signaling axis in human colon carcinoma.^44^ The regulation was later verified to promote c‐Met expression and HGF‐driven c‐Met phosphorylation in HCC cells.^10^ SPINT1, a transmembrane serine protease inhibitor, was reported to inhibit pro‐HGF precursors from becoming activated by forming double‐chain HGF structures.^14^, ^15^, ^16^, ^56^ Here, we used molecular approach to demonstrate that SPINT1 serves as a downstream signaling protein of MACC1, participating in tumor genesis and progression through HGF/c‐Met signaling pathway (Figure S8). Mechanistically, MACC1 overexpression in GC cells promoted the proteolytic cleavage of pro‐HGF and c‐Met phosphorylation by transcriptional inhibiting SPINT1 expressions. Moreover, we speculated that a non‐coding RNA hsa‐miR‐183‐5p may be involved in this regulation. Therefore, the connection between MACC1, SPINT1, and HGF/c‐Met axis shown in this study highlighted the mechanism of TFs function in carcinogenesis and can supplement previous studies on transcriptional regulation.

Although the molecular, pathological, and clinical phenotypes in tumors vary from tissues to organs, some molecular changes are observed to converge into common and general signaling pathways eventually.^49^, ^57^, ^58^, ^59^ HGF/c‐Met signaling axis consisting of HGF and its high‐affinity receptor, c‐Met, is closely associated with the onset, progression, and metastasis of multiple tumors.^49^, ^50^ Although HGF/c‐MET pathway was deemed as a promising therapeutic target, numerous investigations have proven that inhibition of HGF or c‐Met TKI was not an effective and practical therapeutic strategy in suppressing multiple human cancers.^60^, ^61^, ^62^, ^63^ Hence, understanding further the internal mechanism that underlies this pathway regulation was attempted by studying MACC1 and SPINT1 functions. As previous studies reported, the biological functions of MACC1 and SPINT1 can be observed in variety of cancers.^9^, ^13^, ^14^, ^15^, ^45^, ^46^, ^47^ To form a multi‐tumor vision and improve the general applicability, our study commenced at pan‐cancer level. We utilized multiple network databases to screen correlated key TFs in human pan‐cancer repository. Subsequent prognostic assessment demonstrated the validity of MACC1/SPINT1 panel in predicting patient survival among different groups with eight cancer types.

Finding a specific, sensitive, and reliable biomarker has been a long‐term perspective in cancer research and clinical application. Due to the heterogeneity of tumors, a single gene serving as a predictive indicator was dissatisfactory in early diagnosis and patient outcomes.^6^, ^8^, ^64^, ^65^, ^66^ Thus, a combination of correlated or interacting molecules that could serve as a diagnostic and prognostic signature might be an ideal and practical assessment tool for patients with different cancers. Kaplan‐Meier curves and time‐dependent ROC analysis illustrated that the two‐gene signature combining MACC1 with SPINT1 in a linear regress model exhibited better prognostic value for OS and DFS in 128 GC patient samples, in comparison with the MACC1 or SPINT1 expressions separately. Additionally, statistical analysis of eight cancer types in the TCGA database also validated the guaranteeing efficacy of the combined signature. In brief, our study suggested that MACC1/SPINT1 panel can be a reliable prognostic indicator, which provides an advance in tumor prognosis research.

## 5 CONCLUSION

In summary, the MACC1/SPINT1 panel that promotes malignant progression of GC was primarily involved in the regulation of HGF/c‐Met signaling axis. Mechanistically, it was executed by mediating the pro‐HGF proteolysis and c‐Met phosphorylation. Furthermore, we have established the predictive signature model combining MACC1 with SPINT1 for prognosis prediction in patients with GC and other seven cancer types, which will lay a foundation for the development of new biomarkers and targeted therapies in multiple cancer types.

## 6 ETHICS STATEMENT

The use of human tissue specimens and clinical data was approved by the Nanfang Hospital Ethics Review Board (Guangzhou, China). Each subject signed the written informed consent permitted by the Nanfang Hospital Ethics Review Committee (Guangzhou, China) before the study.

## CONFLICT OF INTEREST

The authors have declared that no competing interest exists.

## Supporting information

Fig S1

Fig S2

Fig S3

Fig S4

Fig S5

Fig S6

Fig S7

Fig S8

## Data Availability

The molecular experiment data generated and analyzed during the current study are available from the corresponding author on reasonable request. The bioinformatics analysis data utilized in this study are publicly available and listed below: GEO database (https://www.ncbi.nlm.nih.gov/geo/); TCGA (https://cancergenome.nih.gov); STRING (https://string‐db.org/); SEEK (http://seek.princeton.edu/); Coexpedia (http://www.coexpedia.org/); Oncomine (https://www.oncomine.org); TargetScan (http://www.targetscan.org/); Kaplan–Meier Plotter (www.kmplot.com); cBioPortal (www. cbioportal.org); GEPIA (http://gepia.cancer‐pku.cn/); and UCSC Xena (https://xenabrowser.net/datapages/).
