# Supplementary figures and images for: MET transcriptional regulator/serine peptidase inhibitor kunitz type 1 panel operating through HGF/c‐MET axis as a prognostic signature in pan‐cancer
Source: Cancer Med. 2021 Mar 9;10(7):2442–60. doi: 10.1002/cam4.3834 (PMC7982633; doi:10.1002/cam4.3834)

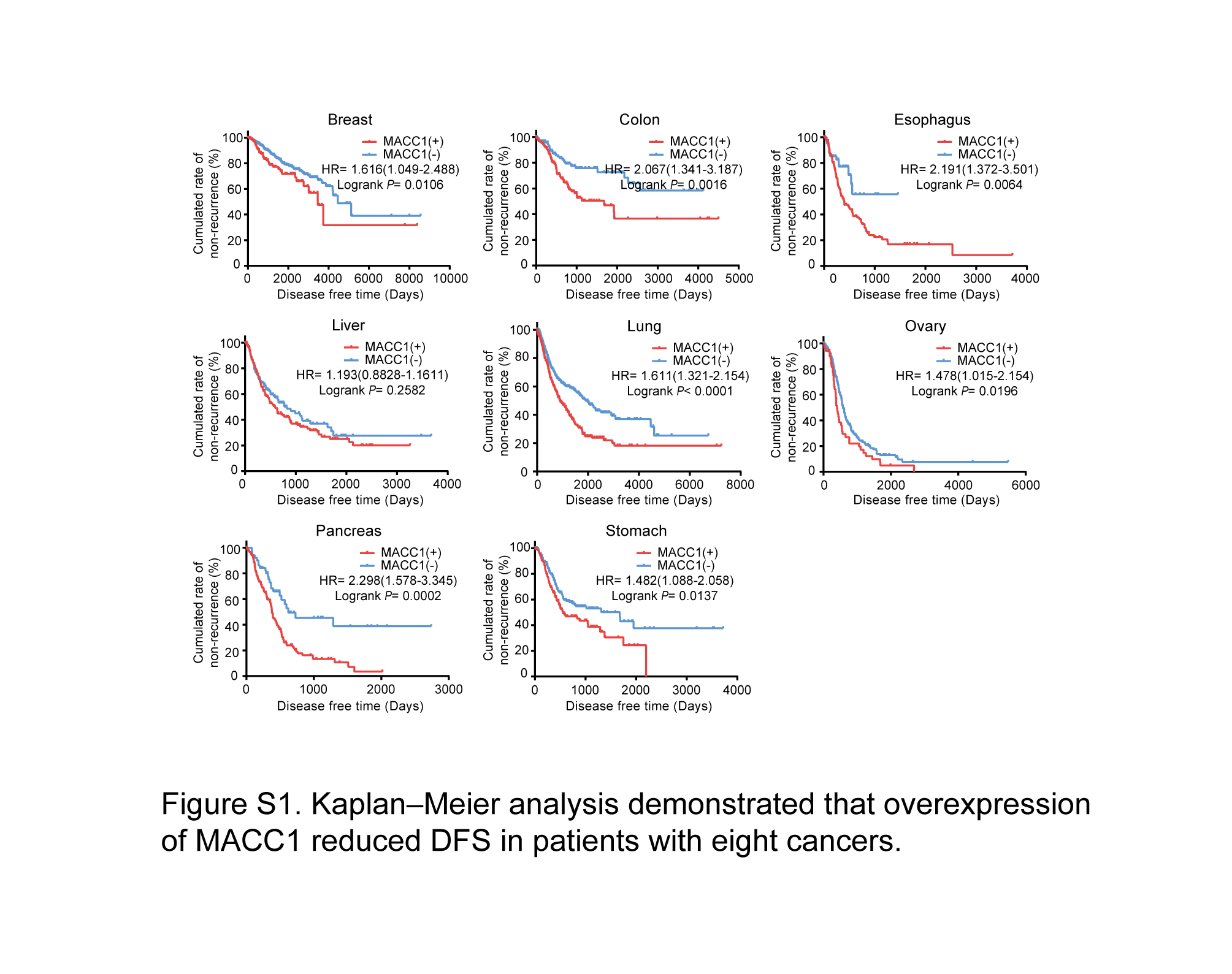

Supplement: Supplementary file 1 — Fig S1 [file CAM4-10-2442-s005.tif]

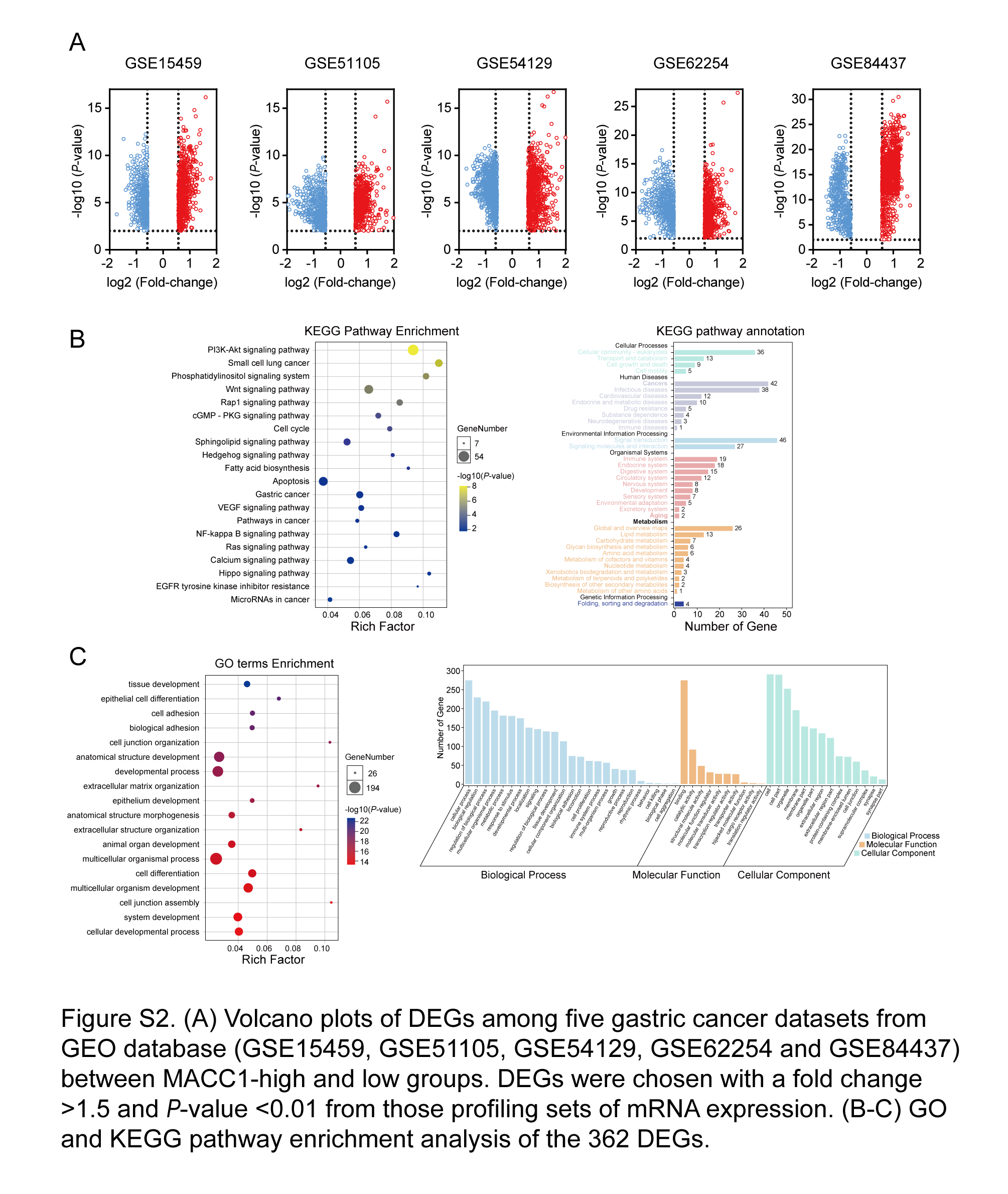

Supplement: Supplementary file 2 — Fig S2 [file CAM4-10-2442-s006.tif]

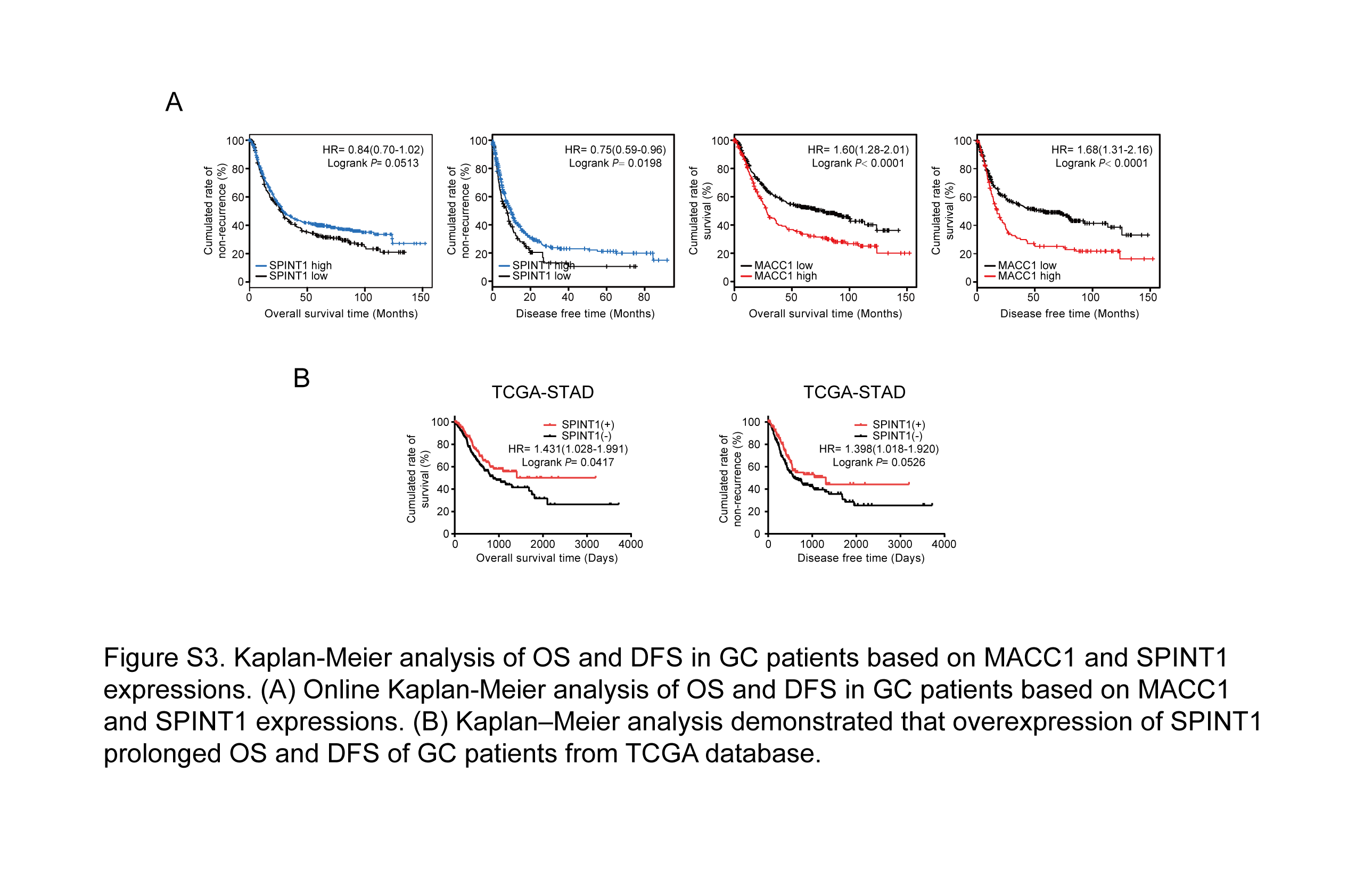

Supplement: Supplementary file 3 — Fig S3 [file CAM4-10-2442-s004.tif]

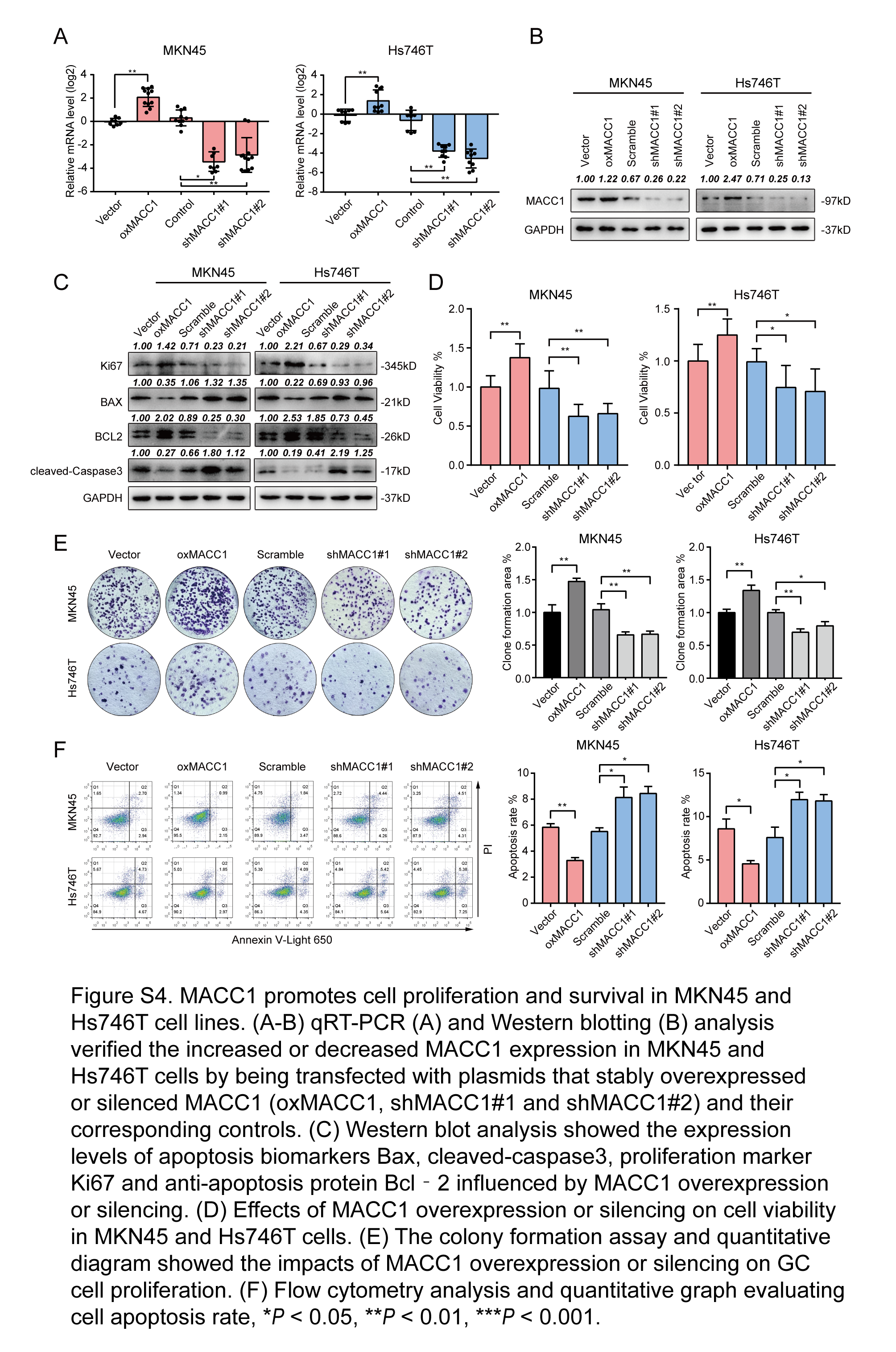

Supplement: Supplementary file 4 — Fig S4 [file CAM4-10-2442-s003.tif]

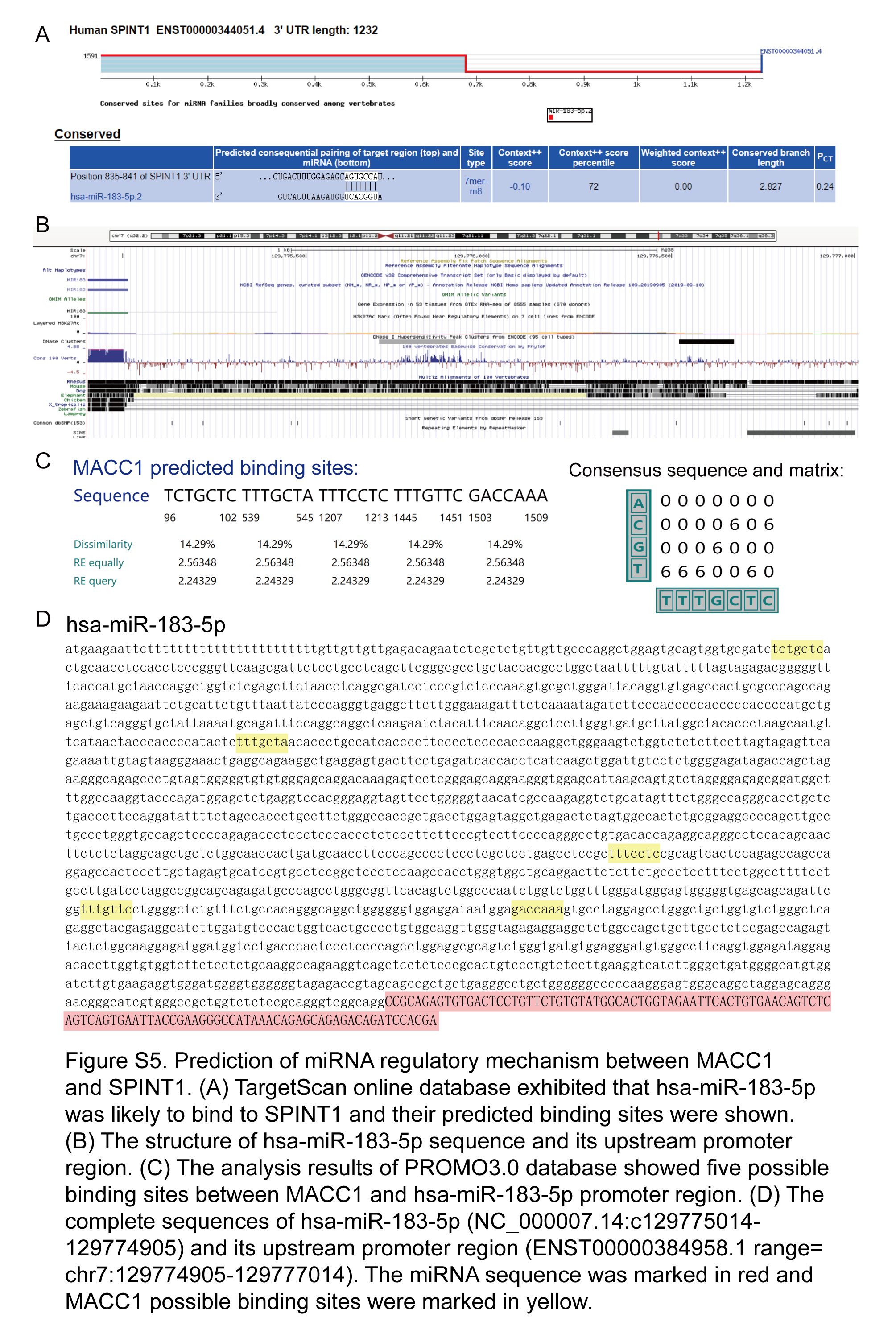

Supplement: Supplementary file 5 — Fig S5 [file CAM4-10-2442-s002.tif]

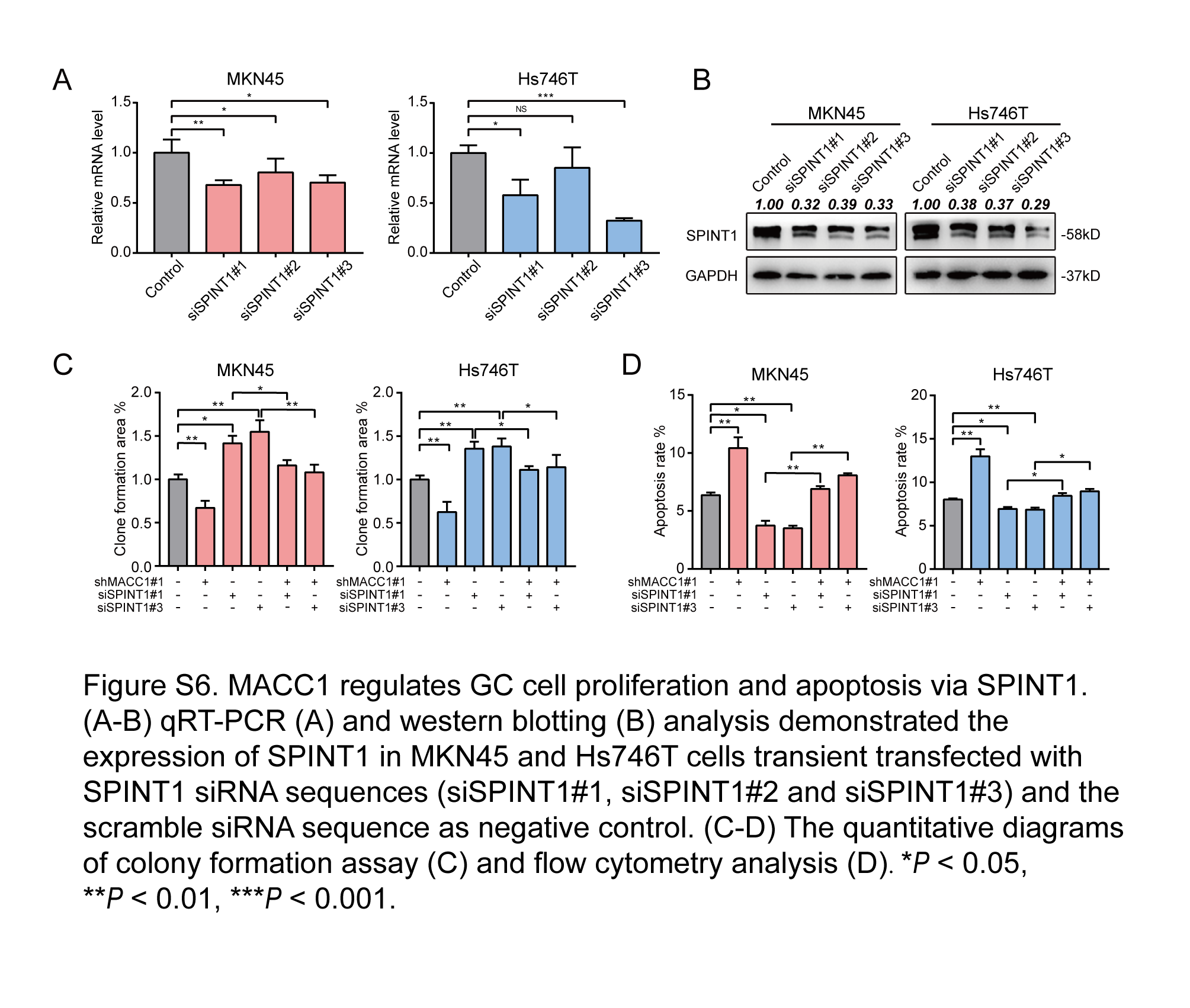

Supplement: Supplementary file 6 — Fig S6 [file CAM4-10-2442-s008.tif]

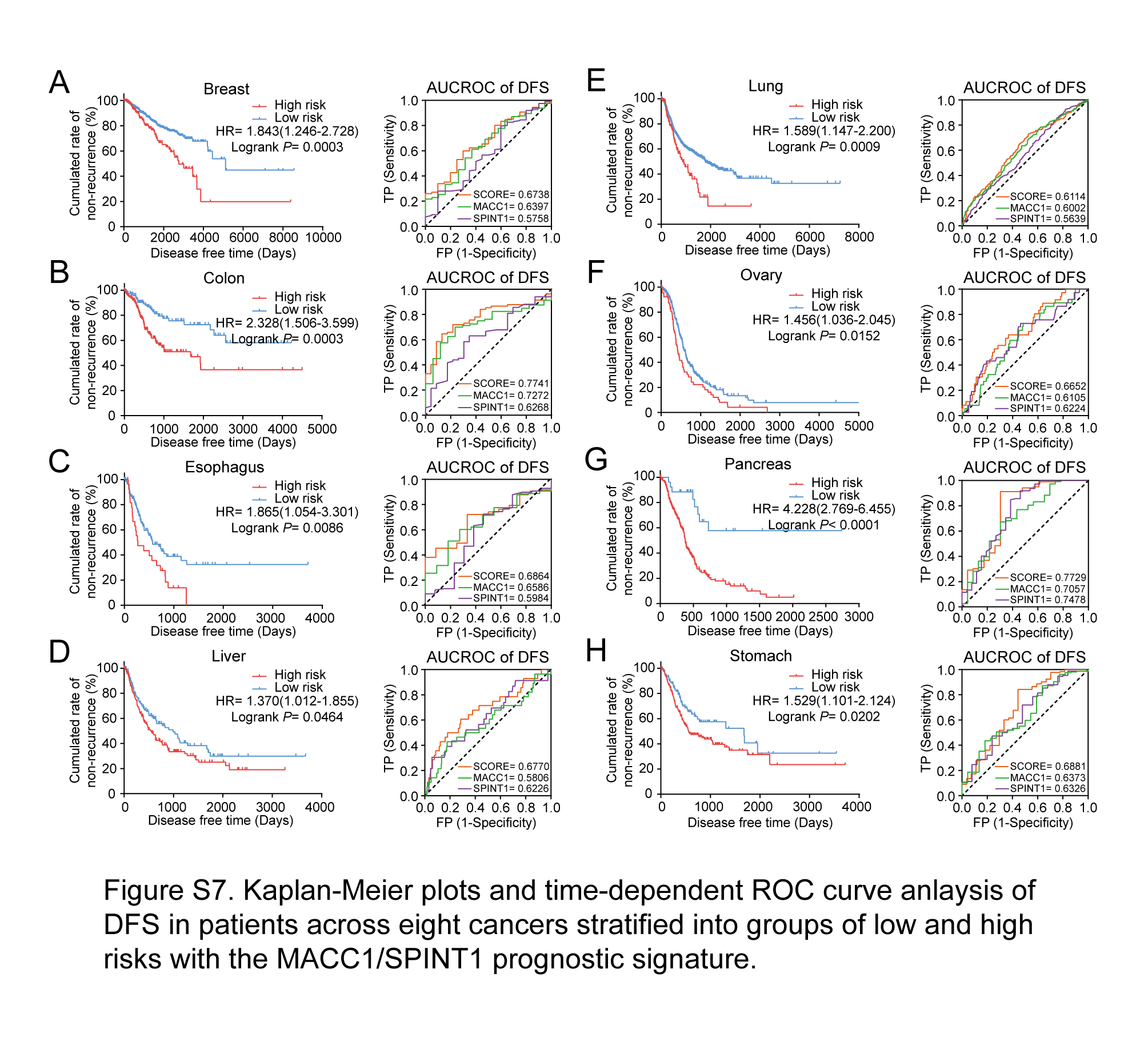

Supplement: Supplementary file 7 — Fig S7 [file CAM4-10-2442-s007.tif]

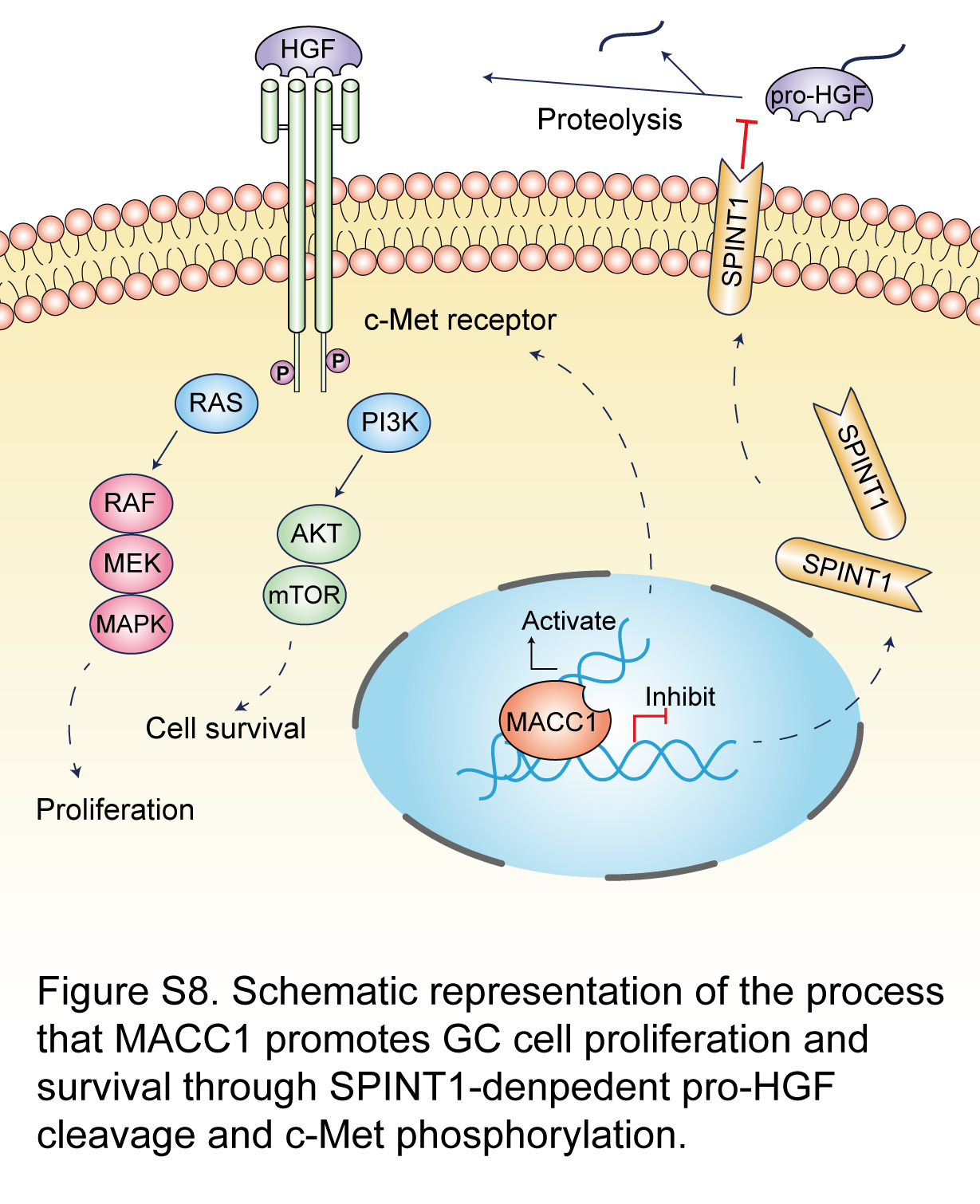

Supplement: Supplementary file 8 — Fig S8 [file CAM4-10-2442-s001.tif]
